# Arterial sheathing in Leber hereditary optic neuropathy

**DOI:** 10.1016/j.ajoc.2022.101431

**Published:** 2022-02-21

**Authors:** Henry W. Zhou, Jeffrey G. Odel

**Affiliations:** Edward S. Harkness Eye Institute, Columbia University Irving Medical Center, New York, NY, USA

**Keywords:** Leber hereditary optic neuropathy, LHON, Arterial vasculitis, Sheathing, Arterial sheathing

## Abstract

**Purpose:**

Presentation of a case of Leber hereditary optic neuropathy (LHON) with arterial sheathing eleven years after initial loss of vision.

**Observations:**

A 46-year-old female was referred for re-evaluation of Leber hereditary optic neuropathy. She first noticed rapid painless loss of vision eleven years prior. Fundus imaging performed at that time did not demonstrate arterial sheathing. Genetic testing revealed the presence of the LHON 11778 G-A mitochondrial mutation. Laboratory values were within normal limits save for angiotensin-converting enzyme elevated to 69 U/L. Eleven years later, visual acuity was count fingers at 12 feet with complete loss of color vision. Funduscopic examination of the optic nerve revealed bilateral pallor, sheathing of the retinal arteries, diffuse vessel narrowing, and tortuous retinal vessels.

**Conclusions and Importance:**

We present a case of LHON that demonstrates retinal arterial sheathing and possibly broadens the spectrum of LHON fundus findings.

## Highlights

• Fundus examination of patients with Leber hereditary optic neuropathy may reveal arterial sheathing.
• Arterial sheathing may appear in the chronic phase of Leber hereditary optic neuropathy.
• There are previous published cases of vascular sheathing in Leber hereditary optic neuropathy.

## 1 Introduction

We present a case of retinal arterial sheathing observed in a LHON patient with the 11778 mutation. Leber hereditary optic neuropathy (LHON) is a maternally inherited mitochondrial disease that causes loss of central vision. Mutations in mitochondrial DNA, the most common being at position 11778,^1^^,^^2^ are necessary but not sufficient for producing optic neuropathy and vision loss.^3^ Fundus findings in early vision loss typically include optic disc hyperemia, peripapillary telangiectasias, vessel tortuosity, and circumpapillary retinal nerve fiber layer (RNFL) swelling.^4^^,^^5^ Optical coherence tomography (OCT) typically reveals RNFL thickening, initially in the temporal and inferior quadrants.^6^ Following vision loss, both the macular thickness and the RNFL undergo thinning.^7^^,^^8^

## 2 Case report

**Fig. 1:**
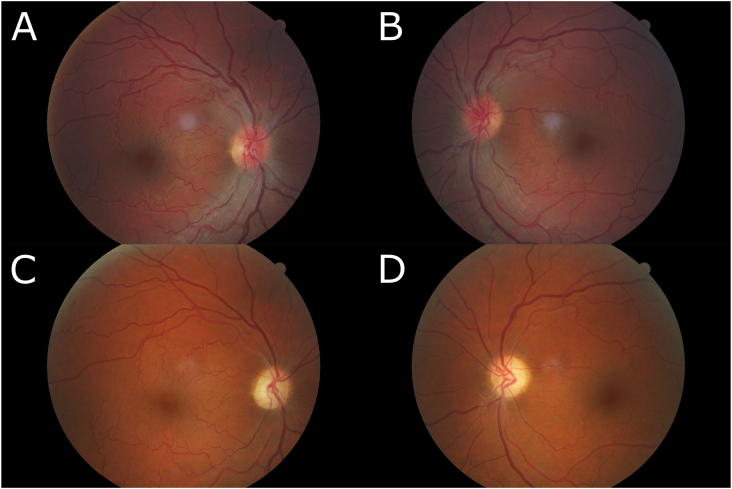
A) Right and B) left eye fundus photographs taken in the acute phase of vision loss demonstrating temporal pallor of both optic discs without evidence of sheathing. C) Right and D) left eye fundus photographs taken eleven years later demonstrating arterial sheathing, vessel narrowing, and scattered telangiectasias.

A 46-year-old female was seen for re-evaluation of Leber hereditary optic neuropathy. She noted acute bilateral painless loss of vision eleven years prior that progressed over 48 hours and stabilized without improvement. Fundus imaging performed at that time did not demonstrate sheathing (Fig. 1A and B). and genetic testing revealed the presence of the LHON 11778 G-A mitochondrial mutation. Fluorescent treponemal antibody absorption test, anti-nuclear antibody, folate, B1, B6, B12, Lyme antibody, thyroid-stimulating hormone, erythrocyte sedimentation rate, hepatic function panel, and basic metabolic panel were unremarkable. Cholesterol was within normal limits at 199 mg/dL. Angiotensin-converting enzyme was slightly elevated to 69 U/L. Chest x-ray was unremarkable. She has no past medical history, and her family history is significant only for glaucoma in her father. She is an active smoker. Her vision slightly progressed since presentation, but her general health remained normal.

On recent exam her visual acuity was counting fingers at 12 feet OU. Her pupils were equal without an afferent pupillary defect. Extraocular movements were full with intact pursuit and saccades. Amsler grid testing showed no evidence of metamorphopsia. She was unable to see any AO/HRR pseudoisochromatic plates. Slit lamp examination revealed clear corneas with no evidence of keratic precipitates and quiet and deep anterior chambers. Her irides had no nodules or synechiae, and her lenses were clear. Intraocular pressure as measured by Goldmann applanation tonometry was 15 mm Hg in both eyes. Funduscopic examination of the optic nerve revealed bilateral marked pallor. The maculae were normal bilaterally. The retinal arteries were sheathed, and her retinal vessels were mildly tortuous with decreased arterial and venous caliber (Fig. 1C and D). She was counseled on the results of her genetic analysis, diagnosis, and prognosis. She was referred to a pulmonologist for follow-up of elevated ACE. Repeat chest x-ray was normal.

## 3 Discussion

LHON must be considered for patients presenting with cryptogenic optic neuropathy, including female patients and patients without family history.^9^ Typical early findings of patients with LHON include optic disc hyperemia, peripapillary telangiectasias, vessel tortuosity, and circumpapillary retinal nerve fiber layer (RNFL) swelling.^4^^,^^5^ One previously described case of sheathing observed in LHON^10^ was published in 2000. In personal communications with LHON experts, there are few such cases of sheathing.

Sheathing of retinal vessels, for retinal arteries and veins alike, is described clinically as retinal vasculitis and may be accompanied by other manifestations including leakage or occlusion.^11^ The mechanism of vascular sheath formation is as of yet unknown. Current hypotheses suggest that type III hypersensitivity and immune complex formation may be involved.^12^ There are numerous known causes of retinal vasculitis, some of which include known causes – toxoplasmosis, cytomegalovirus retinitis, syphilis, diffuse subacute neuroretinitis – and syndromes of unknown causes – Behcet's disease, multiple sclerosis, Eales' disease, sarcoidosis, acute posterior multifocal placoid pigment epitheliopathy, acute zonal occult outer retinopathy, pars planitis, idiopathic recurrent branch retinal artery occlusion, frosted-branch retinal angiitis, acute multifocal hemorrhagic retinal vasculitis, retinal phlebitis and pan-uveitis associated with viral-like upper respiratory disease.^11^^,^^13^, ^14^, ^15^, ^16^, ^17^ Most cases of retinal vasculitis are phlebitis, with a minority of cases demonstrating arteritis. Phlebitis is typically seen in causes such as Behcet's disease, sarcoidosis, tuberculosis, and multiple sclerosis.^12^^,^^18^ Arteritis is typically seen in causes such as systemic lupus erythematosus, polyarteritis nodosa, and acute retinal necrosis.

The inflammatory sheathing observed in the vasculitides described above are distinct from the non-inflammatory sheathing associated with other causes, such as in arteriosclerosis.^19^ There is no current consensus for a term referencing non-inflammatory sheathing, though some authors use the term “pseudo-sheathing.” There is one paper by Nikoskelainen et al. that describes the finding of “pseudo-sheathing” in LHON.^20^ Regardless of terminology, we believe that this observation of arterial sheathing made in the chronic phase of LHON represents non-inflammatory sequelae of the disease, possibly associated with optic atrophy, as opposed to a true vasculitic process. There is also the possibility that the presence of sheathing in chronic LHON represents the presence of a previous inflammatory state.^21^

Herein is a case of LHON with arterial sheathing seen 11 years after visual loss. This case broadens the clinical spectrum of LHON fundus findings to include retinal arterial sheathing in the chronic state.

## 4 Conclusions

This case of LHON demonstrates arterial sheathing, with eleven years follow up after the acute phase.

## Patient consent

Consent to publish the case report was not obtained. This report does not contain any personal information that could lead to the identification of the patient.

## Conflicts of interest

No conflict of interest exists.

## Funding

No funding was received for this work.

## Intellectual property

We confirm that we have given due consideration to the protection of intellectual property associated with this work and that there are no impediments to publication, including the timing of publication, with respect to intellectual property. In so doing we confirm that we have followed the regulations of our institutions concerning intellectual property.

## Research ethics

We further confirm that any aspect of the work covered in this manuscript that has involved human patients has been conducted with the ethical approval of all relevant bodies and that such approvals are acknowledged within the manuscript.

## Authorship

All listed authors meet the ICMJE criteria.  We attest that all authors contributed significantly to the creation of this manuscript, each having fulfilled criteria as established by the ICMJE.

We confirm that the manuscript has been read and approved by all named authors.

We confirm that the order of authors listed in the manuscript has been approved by all named authors.
